# One-carbon metabolizing enzyme ALDH1L1 influences mitochondrial metabolism through 5-aminoimidazole-4-carboxamide ribonucleotide accumulation and serine depletion, contributing to tumor suppression

**DOI:** 10.1038/s41598-023-38142-5

**Published:** 2023-08-18

**Authors:** Masato Sasaki, Kazuo Yamamoto, Takeshi Ueda, Hayato Irokawa, Kouki Takeda, Ryoya Sekine, Fumie Itoh, Yutaka Tanaka, Shusuke Kuge, Nobuyuki Shibata

**Affiliations:** 1 Division of Infection and Host Defense, Faculty of Pharmaceutical Sciences, Tohoku Medical and Pharmaceutical University, 4-4-1, Komatsusima, Aoba-ku, Sendai, Miyagi 981-8558 Japan; 2 Biomedical Research Support Center, Nagasaki University School of Medicine, 1-12-4 Sakamoto, Nagasaki, 852-8523 Japan; 3 Department of Biochemistry, Faculty of Medicine, Kindai University, Osakasayama, Osaka 589-8511 Japan; 4 Faculty of Medicine, Graduate School of Medical Sciences, Kindai University, Osakasayama, Osaka 589-8511 Japan; 5 Division of Microbiology, Faculty of Pharmaceutical Sciences, Tohoku Medical and Pharmaceutical University, 4-4-1, Komatsusima, Aoba-ku, Sendai, Miyagi 981-8558 Japan

**Keywords:** Cancer metabolism, Liver cancer, Nutrient signalling, Experimental models of disease

## Abstract

Tumor cells generally require large amounts of nucleotides, and thus activate de novo purine synthesis (*dn*PS). In the *dn*PS reactions, 10-formyltetrahydorofolate (10-fTHF) supplied by one-carbon metabolism is utilized as a formyl group donor. We focused on aldehyde dehydrogenase 1 family member L1 (ALDH1L1), which metabolizes 10-fTHF to tetrahydrofolate and whose expression is often attenuated in hepatocellular carcinoma (HCC). We generated *ALDH1L1*-expressing HuH-7 cells to perform metabolome analysis and found that intracellular levels of serine were reduced and glycine was increased. In addition, 5-aminoimidazole-4-carboxamide ribonucleotide (ZMP), a *dn*PS intermediate, accumulated due to the consumption of 10-fTHF by ALDH1L1, which inhibited ZMP formylation. Importantly, *ALDH1L1*-expressing cells showed reduced ZMP sensitivity and higher mitochondrial activity. The suppression of mitochondrial serine catabolism by ALDH1L1 expression was speculated to be closely related to this phenotype. Gene set enrichment analysis utilizing The Cancer Genome Atlas data revealed that genes related to oxidative phosphorylation were enriched in HCC patients with high *ALDH1L1* expression. Moreover, drug sensitivity data analysis demonstrated that HCC cell lines with low expression of *ALDH1L1* were sensitive to ZMP and cordycepin, a structural analog of ZMP and AMP. Our study revealed that ZMP and AMP analogs might be effective in the pharmacotherapy of HCC patients with low expression of *ALDH1L1*.

## Introduction

According to recent epidemiological studies, primary liver cancer is the sixth most common cancer type and the third most common cause of cancer death worldwide^1^. In most countries, hepatocellular carcinoma (HCC) accounts for approximately 75% of all liver cancers^2^. Primary liver cancer is known to develop from chronic liver diseases such as hepatitis B and C, non-alcoholic fatty liver disease, and non-alcoholic steatohepatitis (NASH), and risk factors include excessive alcohol consumption, obesity, and diabetes^3^. Although attempts are being made to treat HCC through the use of molecular targeted drugs such as sorafenib and personal genome analysis by next-generation sequencing, mean survival is 6–20 months^4^ and the 5 years survival rate is approximately 10%^5^. Therefore, many problems remain to be resolved to prolong the survival period of patients with HCC and to cure them.

It is well known that cancer cells including HCC cells require a large number of nucleotides for their proliferation. Among these nucleotides, purine nucleotides are supplied by the salvage pathway or de novo synthesis. In de novo purine nucleotide synthesis, inosine monophosphate (IMP) is synthesized from 5-phosphoribosyl 1-pyrophosphate (PRPP), which is generated from the pentose phosphate pathway, through a cascade of 10 reactions. These reactions are catalyzed by six enzymes and require glycine (Gly), glutamine, aspartic acid, and 10-formyltetrahydorofolate (10-fTHF). 10-fTHF is used to synthesize formylglycinamide ribonucleotide (FGAR) from glycinamide ribonucleotide (GAR) and 5-formamidoimidazole-4-carboxamide ribonucleotide (FAICAR) from 5-aminoimidazole-4-carboxamide ribonucleotide (ZMP, also known as AICAR) by transferring formyl groups, and these reactions are catalyzed by phosphoribosylglycinamide formyltransferase (GART) and 5-aminoimidazole-4-carboxamide nucleotide formyltransferase/IMP cyclohydroxylase (ATIC), respectively^6^. ZMP, a purine synthesis intermediate, is an analog of AMP and is known to function as an endogenous AMP-activated protein kinase (AMPK) activator^7^. AMPK activation generally regulates cellular energy metabolism by activating catabolic pathways and inhibiting anabolic pathways^7,8^. 10-fTHF is supplied by folate metabolism, which is also called one-carbon metabolism. One-carbon metabolism is involved in de novo purine synthesis (*dn*PS) as well as thymidylate synthesis, formyl-methionine synthesis, and *S*-adenosylmethionine synthesis in conjunction with the methionine (Met) cycle^9^. Thus, *dn*PS is closely related to one-carbon metabolism and is expected to have a significant impact on cellular energy metabolism.

In the cytosol, serine hydroxymethyltransferase 1 (SHMT1) converts tetrahydrofolate (THF) and serine (Ser) to 5,10-methylenetetrahydrofolate (5,10-CH_2_-THF) and Gly. 5,10-CH_2_-THF is metabolized to 5-methyltrahydrofolate (5-CH_3_-THF) by 5,10-methylenetetrahydrofolate reductase (MTHFR) or to 5,10-methenyltetrahydrofolate (5,10-CH=THF) by methylenetetrahydrofolate dehydrogenase 1 (MTHFD1), which also catalyzes 5,10-CH=THF to 10-fTHF. Cytosolic 10-fTHF is metabolized by two enzymes in addition to ATIC and GART. MTHFD1 uses 10-fTHF as a substrate to produce THF and formate in a reversible reaction. Another enzyme is aldehyde dehydrogenase 1 family member L1 (ALDH1L1), which converts 10-fTHF and NADP^+^ to THF, NADPH, and CO_2_. As well as cytoplasm, folate-metabolizing enzymes exist in mitochondria. SHMT2, like SHMT1, catalyzes THF and Ser to 5,10-CH_2_-THF and Gly. In addition, THF to 5,10-CH_2_-THF conversion is also accomplished by the glycine cleavage system in mitochondria. The conversion of 5,10-CH_2_-THF to 5,10-CH=THF and 5,10-CH=THF to 10-fTHF is performed by NAD^+^-dependent MTHFD2 or NADP^+^-dependent MTHFD2L. MTHFD1L contributes to the reversible production of THF and formic acid from 10-fTHF, as does cytosolic MTHFD1. Furthermore, ALDH1L2 performs a similar reaction to ALDH1L1 in mitochondria. It is thought that there is no enzymatic translocation between the cytoplasm and mitochondria, and that mutual metabolism is balanced by the transfer of THF, formate, Ser, and Gly^9–11^.

Among these groups of enzymes involved in one-carbon metabolism, we focused on ALDH1L1 because *ALDH1L1* is relatively highly expressed in the liver and its expression has been reported to be decreased in liver cancer^12–16^. In addition, many studies have predicted that *ALDH1L1* is a tumor suppressor gene^17^. In fact, analyses of recently generated *Aldh1l1*-deficient mice have shown that *Aldh1l1* deficiency significantly affects intracellular metabolism, including one-carbon metabolism, and that this deficiency greatly accelerates tumor incidence in a diethylnitrosamine-induced liver carcinoma mouse model^18–20^. In this study, to understand the role of ALDH1L1 in tumor suppression, we expressed *ALDH1L1* in HuH-7 cells, which have reduced *ALDH1L1* expression, and compared the effects of ALDH1L1 expression in HuH-7 cells. We found that endogenous ZMP that accumulated as a result of *ALDH1L1* expression restricted oxidative phosphorylation (OXPHOS). It was suggested that *ALDH1L1* expression altered mitochondrial Ser catabolism. *ALDH1L1* expression also decreased ZMP sensitivity, indicating the efficacy of ZMP or related AMP analogs in the treatment of HCC with reduced *ALDH1L1* expression.

## Results

### Increased one-carbon metabolism by *ALDH1L1* expression leads to decreased Ser and increased Gly and ZMP

According to the Human Protein Atlas database^21^, among 24 liver cancer cell lines, HepG2, HuH-1, HuH-6, and SNU-878 cells expressed ALDH1L1, while the others had no or quite low expression. Because it was difficult to compare expression levels with normal tissues based on these database results alone, we compared *ALDH1L1* expression levels in 20 cell lines, including 2 liver cancer cell lines, using normal tissue-derived pooled cDNA as a reference (Supplemental Fig. 1A). HuH-7 and HepG2 cells exhibited low and high *ALDH1L1* expression, respectively. These data do not contradict the Human Protein Atlas database. To mimic the different *ALDH1L1* expression states of human HCC, we generated control (H7-emp) or *ALDH1L1* (1L1)-expressing HuH-7 (H7-1L1) cells using lentivirus vectors (Supplemental Fig. 1B). Cell proliferation and cellular morphology under normal conditions did not differ between H7-emp and H7-1L1 cells (Supplemental Fig. 1C). Consistent with the fact that *Aldh1l1* deficient mice have no effect on growth or lifespan under normal rearing conditions^18^, *ALDH1L1* expression probably has no effect on cell proliferation rates.

**Figure 1:**
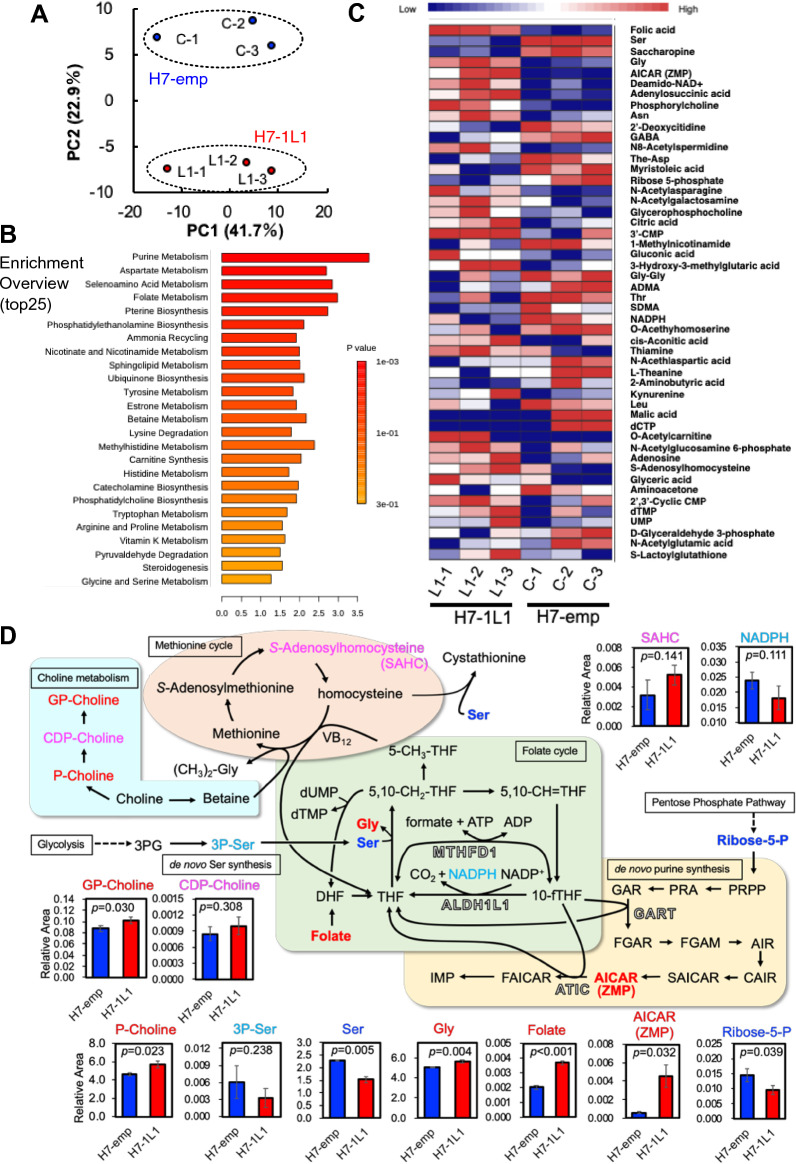
Summary of metabolome analysis of H7-emp and H7-1L1 cells. (**A**) PCA of H7-emp and H7-1L1 cells (*n* = 3). (**B**) Metabolite set enrichment analysis (MSEA) of the top 25 metabolic pathways. (**C**) Heatmap shows relative abundance patterns of 50 metabolites with significantly different abundance between H7-emp and H7-1L1 cells. (**D**) Significantly and relatively upregulated metabolites in H7-emp cells are indicated in blue and cyan, respectively. Furthermore, significantly and relatively upregulated metabolites in H7-1L1 cells are shown in red and magenta, respectively. Four cytosolic enzymes that utilize 10-fTHF as a substrate are indicated by boxed characters. Results are the mean ± SEM of triplicates; *P*-values, unpaired Student’s *t*-test. Abbreviations: 3′-CMP, 3′-cytidine monophosphate; 3PG, 3-phosphoglyceric acid; 3P-Ser, 3-phosphoserine; ADMA, asymmetric dimethylarginine; AICAR/ZMP, 5-aminoimidazole-4-carboxamide ribonucleotide; AIR, 5-aminoimidazole ribonucleotide; ATIC, 5-aminoimidazole-4-carboxamide nucleotide formyltransferase/IMP cyclohydroxylase; CAIR, carboxyaminoimidazole ribonucleotide; CDP, cytidine diphosphate; dCTP, deoxycytidine triphosphate; DHF, dihydrofolate; dTMP, deoxythymidine monophosphate; dUMP, deoxyuridine monophosphate; FAICAR, 5-formamidoimidazole-4-carboxamide ribonucleotide; FGAM, formylglycinamidine; FGAR, formylglycinamide ribonucleotide; GABA, gamma-aminobutylic acid; GAR, glycinamide ribonucleotide; GART, phosphoribosylglycinamide formyltransferase; GP-choline, glycerophosphocholine; IMP, inosine monophosphate; MTHFD1, cytosolic methylenetetrahydrofolate dehydrogenase; NADP^+^, nicotinamide adenine dinucleotide phosphate oxydized; NADPH, nicotinamide adenine dinucleotide phosphate reduced; P-choline, phosphorylcholine; PRPP, phosphoribosyl pyrophosphate; SAICAR, phosphoribosyl aminoimidazole succinocarboxamide; SDMA, symmetric dimethylarginine; THF, tetrahydrofolate; UMP, uridine monophosphate; VB_12_, vitamin B_12_.

To understand the direct metabolic changes resulting from *ALDH1L1* expression, we measured the amounts of intracellular metabolites in both H7-emp and H7-1L1 cells by CE-TOFMS analysis. A principal component analysis (PCA) plot indicated that a clear difference was observed in the metabolome profile (Fig. 1A). To identify significantly altered metabolic pathways, we performed metabolite set enrichment analysis (MSEA) and pathway analysis (https://www.metaboanalyst.ca/). Notably, purine and folate metabolism were highly enriched, while other energy metabolism mechanisms such as OXPHOS were not (Fig. 1B). These related metabolites were included in the top 50 influenced metabolites (Fig. 1C). Based on these analyses, actual major metabolite alterations are depicted in Fig. 1D. Because ALDH1L1 is a folate metabolism enzyme, we predicted that folate intermediates would be metabolically influenced. Unfortunately, we could not detect any folate intermediates, except for a folate that was abundant in H7-1L1 cells. Importantly, Ser was significantly reduced, while Gly was increased in H7-1L1 cells. These data suggest that cytosolic THF to 5,10-CH_2_-THF conversion was promoted because Ser consumption and Gly production occur in this reaction. Consistent with these observations, the de novo Ser synthesis precursor, phospho-serine, was also marginally reduced in H7-1L1 cells. It is known that 5,10-CH_2_-THF is metabolized into three different folate metabolites, dihydrofolate (DHF), 5-CH_3_-THF, and 10-fTHF. 5-CH_3_-THF donates a methyl group to homocysteine, a component of the Met cycle, and is utilized for Met synthesis cooperatively with cobalamin (vitamin B_12_). This reaction was presumably enhanced in H7-1L1 cells because *S*-adenosylhomocysteine, a metabolic intermediate of the Met cycle, tended to increase. It is also known that homocysteine accepts a methyl group from betaine, which is generated from choline and is converted to Met. However, phosphocholine and glycerophosphocholine, which are also produced from choline, were remarkably increased in H7-1L1 cells. Therefore, it was assumed that betaine did not significantly contribute to the Met cycle, and 5-CH_3_-THF was largely involved.

Cytosolic 10-fTHF is catalyzed not only by ALDH1L1 and MTHFD1 in the folate cycle, but also by GART and ATIC in *dn*PS. Interestingly, ZMP was significantly accumulated in H7-1L1 cells. It is suggested that high levels of ZMP resulted from 10-fTHF reduction due to ALDH1L1 expression.

### ALDH1L1 increases the cellular metabolic activity in an AMPK-independent manner

Because ZMP is an endogenous multifunctional metabolite, we focused on the effect of ZMP in H7-emp and H7-1L1 cells. It is known that 5-aminoimidazole-4-carboxamide ribonucleoside (AICAr) is phosphorylated by adenosine kinase and converted into ZMP^22^, we examined adenosine kinase expression between H7-emp and H7-1L1 cells. Western blot analysis revealed that adenosine kinase protein expression was comparable (Supplemental Fig. 2A). In addition, the activity of adenosine kinase is known to be altered by metabolites such as adenosine, AMP, ADP and ATP^23^. However, no differences were found in the amounts of these metabolites (Supplemental Fig. 2B). Thus, the adenosine kinase activities of both H7-emp and H7-1L1 cells are comparable, suggesting that exogenous AICAr may be converted to ZMP to the same extent.

**Figure 2:**
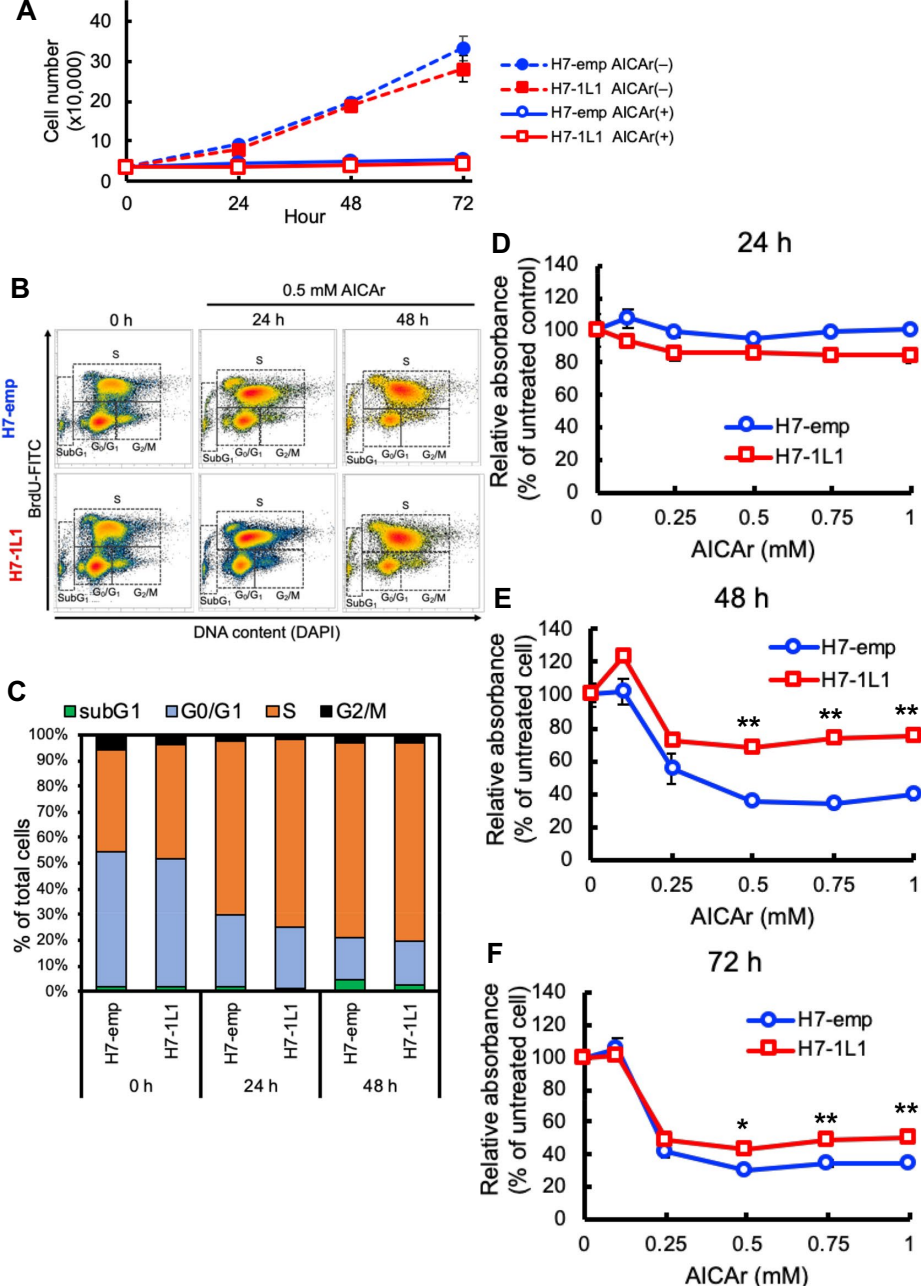
AICAr induced cell cycle arrest and reductase activity of MTS compound. (**A**) Proliferation curves of H7-emp and H7-1L1 cells cultured in 0.5 mM AICAr or vehicle (PBS(−))-containing medium. Results are the mean ± SEM of triplicates. (**B**, **C**) BrdU-labeled cell cycle analysis of H7-emp and H7-1L1 cells treated with 0.5 mM AICAr for indicated periods. The cells were prepared and stained with BrdU and DAPI for fluorescence-activated cell sorting analysis (**B**). Percentages of cells in each cell cycle phase (**C**). (**D**–**F**) MTS analysis of H7-emp and H7-1L1 cells treated with AICAr for 24 h (**D**), 48 h (**E**), and 72 h (**F**). Results are the mean ± SEM; *P < 0.01, **P < 0.001, compared with H7-emp cells, unpaired Student’s *t*-test.

Cells were treated with AICAr for 24, 48, and 72 h, and then the cell number was counted. AICAr vigorously suppressed cell proliferation and arrested the cell cycle in both H7-emp and H7-1L1 cells (Fig. 2A). Combined with BrdU incorporation and DNA content analysis, the contribution of the cell cycle was estimated. AICAr clearly arrested the cell cycle in S phase, but the contribution of each cell cycle phase was comparable between H7-emp and H7-1L1 cells (Fig. 2B,C). Consistent with the results of cell counting and cell cycle analysis, H7-emp cells showed an AICAr concentration-dependent decrease in relative absorbance values at 48 and 72 h when cellular metabolic activity was measured by the MTS assay. On the other hand, relative absorbance values were significantly higher in H7-1L1 cells at 48 and 72 h compared to H7-emp (Fig. 2D–F). It is known that ZMP activates AMPK by binding to regulatory γ subunits. There is a possibility that endogenously accumulated ZMP and exogenously treated AICAr activate AMPK and then cause this phenotype. Thus, we assessed the phosphorylation status of threonine 172 in AMPKα subunits by immunoblotting analysis (Supplemental Fig. 3). Compared with the H7-emp cells, AMPK phosphorylation was not enhanced in H7-1L1 cells despite the accumulation of ZMP. When AICAr was added for 24 or 48 h, moderate phosphorylation of AMPK was observed, whereas strong AMPK activation was observed following addition of AMPK activator A-769662 alone or co-treatment with AICAr. Even in both conditions, there was no difference in AMPK activation between H7-emp and H7-1L1 cells. Similar results were obtained for the phosphorylation status of acetyl-CoA carboxylase (ACC), which is a direct substrate of AMPK. Moreover, MTHFD1, GART, and ATIC expression were not influenced by *ALDH1L1* expression. These data indicated that AICAr resistance observed in H7-1L1 cells is an AMPK-independent event.

### ALDH1L1 sustains mitochondrial activity by modulating electron transport chain function and proton leak in response to AICAr

**Figure 3:**
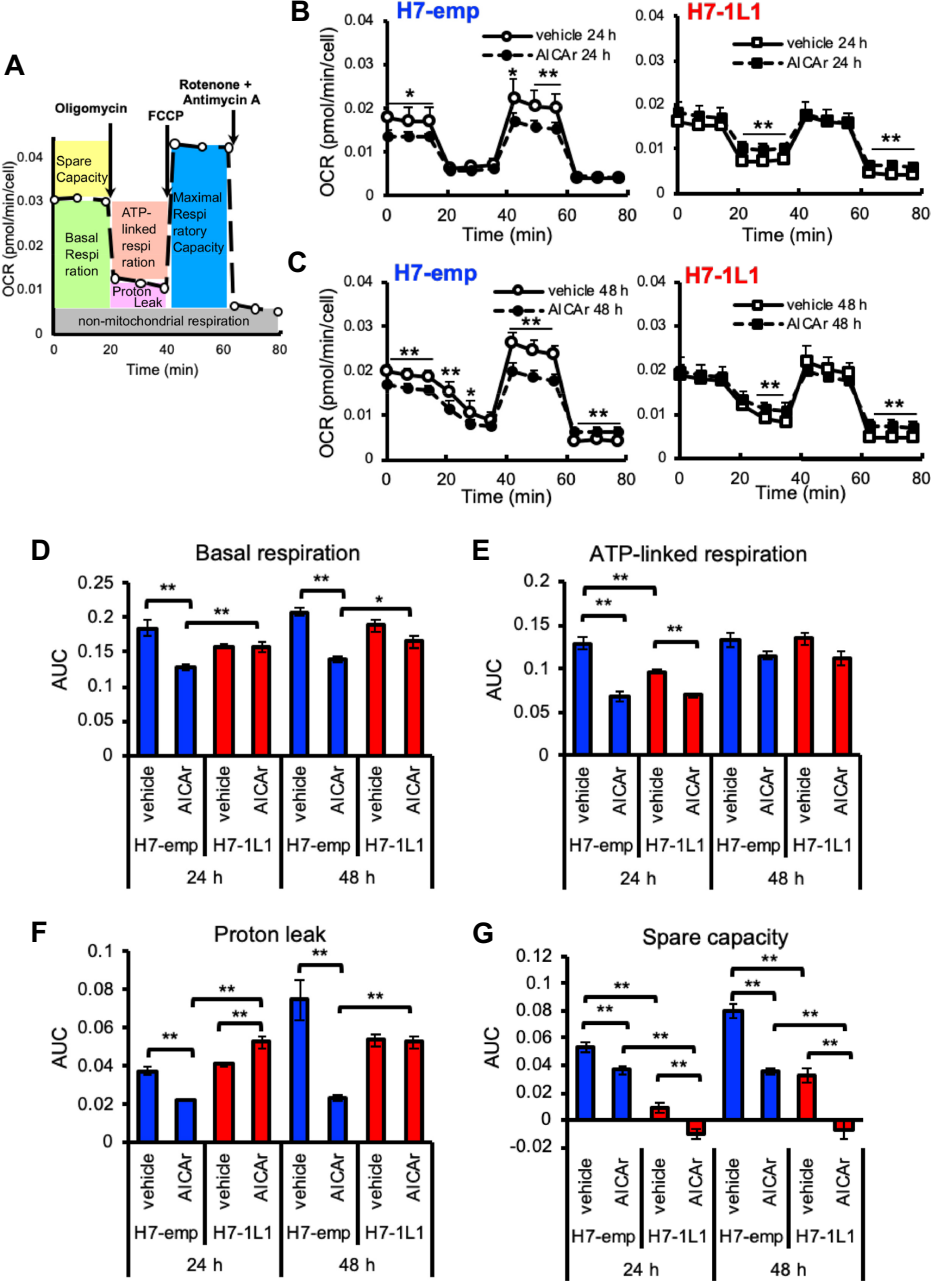
AICAr-induced OCR alterations. (**A**) Schematic representation of the mitochondrial stress test. (**B, C**) Two representative OCR traces of vehicle and 0.5 mM AICAr treatment in response to oligomycin, FCCP, rotenone, and antimycin A in H7-emp cells (left) and H7-1L1 cells (right) for 24 h (B) and 48 h (C) (*n* = 8). (**D-G**) Statistical analysis of the differences in basal respiration (D), ATP-linked respiration (E), proton leakage (F), and spare capacity (G) (*n* = 8). Results are the mean ± SEM; *P < 0.01, **P < 0.001, unpaired Student’s *t*-test.

The MTS assay is based on the reduction of the MTS compound by an enzyme(s) that is assumed to be mitochondrial succinate-tetrazolium reductase. Thus, we hypothesized that AICAr resistance by ALDH1L1 expression depends on mitochondrial activity. To test this possibility, H7-emp and H7-1L1 cells were submitted to mitochondrial oxygen consumption rate (OCR) determination using a cell flux analyzer, which is able to estimate basal respiration, ATP-linked respiration, proton leak, non-mitochondrial respiration, and spare capacity (Fig. 3). Kinetic profiling revealed that H7-emp cells were more highly affected by 24- and 48 h AICAr treatment than H7-1L1 cells (Fig. 3B,C). Indeed, AICAr induced a significant decrease in basal respiration and proton leak in H7-emp cells but not in H7-1L1 cells (Fig. 3D,F). Compared with vehicle-treated cells, AICAr significantly attenuated ATP-linked respiration in both H7-emp and H7-1L1 cells at 24 h and slightly at 48 h. (Fig. 3E). Because basal respiration is the sum of ATP-linked respiration and proton leak, these data suggest that H7-1L1 cells maintained mitochondrial respiration by sustaining the proton leak in the presence of AICAr. Uncoupling protein (UCP) is one of the main factors of proton leak, but the expression level of UCP2, which is known to be ubiquitously expressed, was comparable between H7-emp and H7-1L1 cells regardless of the presence of AICAr (Supplemental Fig. 2). These data suggest that proton leak is regulated by UCP2 activation or other factors. The spare capacity, which is probably related to mitochondrial proton gradient produced by the electron transport chain (ETC) complex, was reduced in H7-1L1 cells regardless of the presence of AICAr, suggesting that ALDH1L1 indirectly suppressed ETC. However, H7-emp and H7-1L1 cells showed reduced spare capacity by AICAr at comparable rates, indicating ETC was inhibited by AICAr (Fig. 3G).

**Figure 4:**
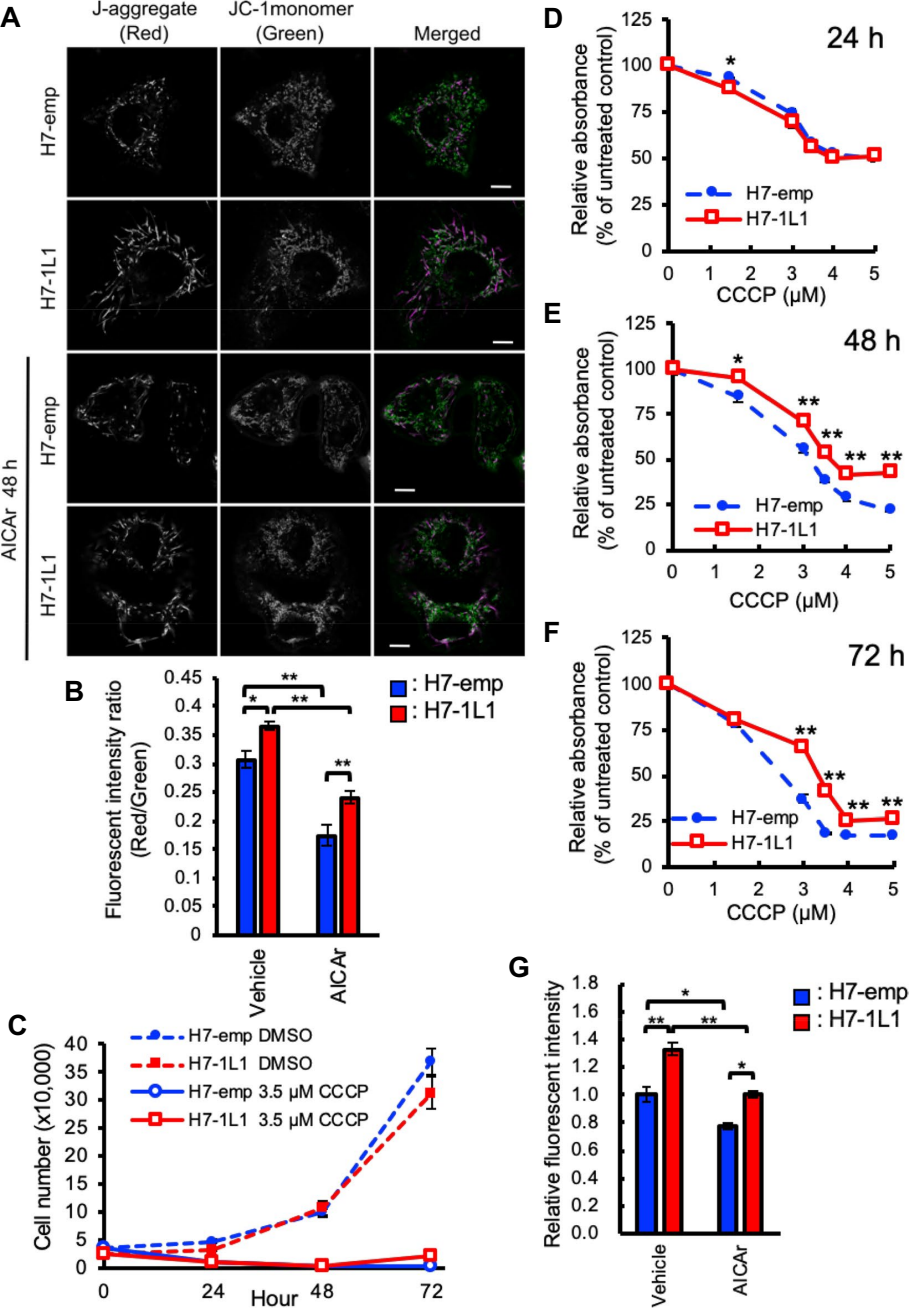
Assessment of mitochondrial activity. (**A**) Live cell images of J-aggregate and JC-1 monomer in H7-emp and H7-1L1 cells treated vehicle or 0.5 mM AICAr for 48 h. Scale bar, 10 µm. (**B**) Statistical analysis of the fluorescent intensity ratio of J-aggregate and JC-1 monomer in H7-emp and H7-1L1 cells treated with vehicle or 0.5 mM AICAr for 48 h. Results are the mean ± SEM; *P < 0.03, **P < 0.001, unpaired Student’s *t*-test. (**C**) Proliferation curves of H7-emp and H7-1L1 cells grown in 3.5 µM CCCP- or vehicle (DMSO)-containing medium. Results are the mean ± SEM of triplicates. (**D–F**). MTS analysis of H7-emp and H7-1L1 cells treated with CCCP for 24 h (D), 48 h (E), and 72 h (F). Results are the mean ± SEM; *P < 0.03, **P < 0.001, compared with H7-emp cells, unpaired Student’s *t*-test. (**G**) Relative mitochondrial ROS levels in H7-emp and H7-1L1 cells treated with vehicle or 0.5 mM AICAr for 48 h. Results are the mean ± SEM; *P < 0.01, **P < 0.001, unpaired Student’s *t*-test.

To support this idea, JC-1 dye was utilized to assess mitochondrial activity. It is known that JC-1 monomers are incorporated into mitochondria and form aggregates that are called J-aggregates. When the mitochondrial membrane is depolarized, JC-1 monomers emit green fluorescence. However, J-aggregate formation is facilitated when the mitochondrial membrane is polarized and emits red fluorescent. Therefore, the red/green fluorescence ratio could estimate mitochondrial membrane potential, which is used as an indicator of cellular health status. In normal growth conditions, red fluorescence from J-aggregates was stronger in H7-1L1 cells than in H7-emp cells. Although AICAr attenuated both green and red fluorescence, similar results were obtained (Fig. 4A). The red/green fluorescence ratio indicated that ALDH1L1 expression resulted in high mitochondrial membrane potential (Fig. 4B).

It seemed contradictory that the spare capacity was reduced in H7-1L1 cells despite their higher mitochondrial membrane potential. In H7-1L1 cells, proton leak was sustained even with AICAr treatment, while the spare capacity was dramatically reduced (Fig. 3F,G). These data suggest that the proton gradient caused by ETC was offset by proton leak through UCPs rather than ATP synthase. In other words, ETC was not completely inhibited by *ALDH1L1* expression and/or AICAr. To verify this possibility, we analyzed the cellular response of carbonyl cyanide 3-chlorophenylhydrazone (CCCP), which is a protonophore mitochondrial uncoupler. Compared with DMSO control, CCCP strongly inhibited proliferation in both H7-emp and H7-1L1 cells (Fig. 4C). Conversely, in the MTS assay, H7-1L1 cells exhibited higher formazan production than H7-emp cells (Fig. 4D–F). These data suggest that mitochondrial succinate-tetrazolium reductase is moderately activated despite CCCP-induced mitochondrial depolarization. Because mitochondrial succinate-tetrazolium reductase might partly participate in ETC, ALDH1L1 partially inhibits proton gradient formation by ETC but appear to retain succinate-tetrazolium reductase activity. Mitochondrial reactive oxygen species (ROS) were higher in H7-1L1 cells than H7-emp cells with or without AICAr because ETC is a major source of ROS^22^ (Fig. 4G). These observations are consistent with the higher mitochondrial membrane potential in H7-1L1 cells.

### Liver cancer cell lines with low ALDH1L1 expression exhibit ZPM and cordycepin sensitivity, and OXPHOS-related genes are enriched in ALDH1L1-expressing patients

**Figure 5:**
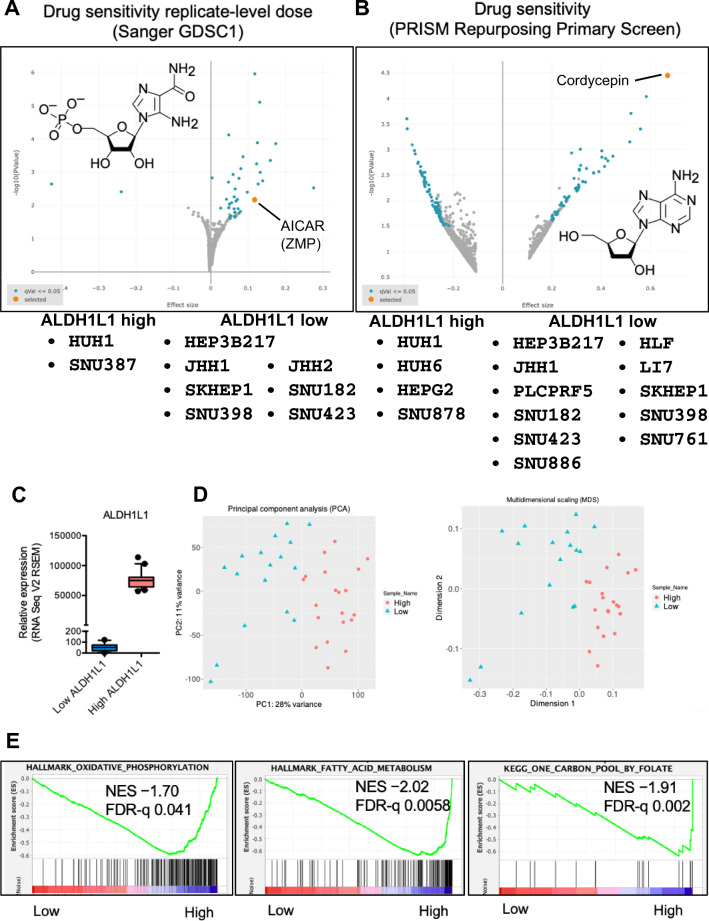
Drug sensitivity in liver cancer cell lines and gene expression in patients with hepatocellular carcinoma. (**A**, **B**) Volcano plot showing the correlation between *ALDH1L1* gene expression and drug response in liver cancer cell lines from the Sanger GDSC1 drug sensitivity replicate-level dosing data set (**A**), and the drug sensitivity PRISM repurposing primary screen (**B**). Molecular structures of AICAr and cordycepin are depicted in each panel. (**C**) Box plot showing relative mRNA expression levels (RSEM counts) of *ALDH1L1* of TCGA hepatocellular carcinoma in cases with low *ALDH1L1* (n = 17, Z-score < − 1.2) versus those with high *ALDH1L1* expression (n = 20, Z-score > 1.2). The boxes extend the 25^th^–75th percentiles; the line in the middle of the box represents the median value, the whiskers represent the 10^th^–90th percentiles, and the outliers are shown as dots. Gene expression data were obtained from TCGA PanCancer Atlas. (**D**) Principal component analysis (PCA) and multidimensional scaling (MDS) of gene expression profiles in 37 hepatocellular carcinoma cases shown in (**C**). Gene expression levels were analyzed as logarithmic values. Sample Name: high, cases with high *ALDH1L1* expression; low, cases with low *ALDH1L1* expression. Cases with low and high *ALDH1L1* expression are segregated by the gene expression profiles of the two groups. (**E**) GSEA plot showing significantly decreased expression of genes associated with oxidative phosphorylation (left), fatty acid metabolism (middle), and one-carbon pool metabolism by folate (right) pathways in patients with low *ALDH1L1* expression compared with those with high *ALDH1L1* expression shown in (**C**).

To examine whether our findings are dependent on *ALDH1L1* expression levels or the intrinsic features of HuH-7 cells, we utilized the cancer dependency map (DepMap) tool and analyzed the drug sensitivity of liver cancer cell lines. We categorized the liver cancer cell lines into two groups of high and low *ALDH1L1* mRNA expression in the public 22Q4 expression data set [high log2 (TPM + 1) > 1.5; low log2 (TPM + 1) < 1.0]. Using the Sanger GDSC1 data set, we showed that ZMP was a sensitive compound in the low *ALDH1L1* expression group [q-value 0.0225] (Fig. 5A). Therefore, there is a possibility that not only HuH-7 cells but also other cancer cell lines respond to ZMP depending on the expression level of *ALDH1L1*. Interestingly, in the PRISM repurposing primary screen data set, cordycepin, which is also an AMP analog, was the most sensitive compound in the low *ALDH1L1* expression group [*q*-value < 0.0001] (Fig. 5B). These findings suggest that purine analogs might be efficient against HCC with low or no *ALDH1L1* expression.

Finally, we analyzed HCC patient datasets in TCGA. Patient data were divided into two groups according to *ALDH1L1* expression levels (Fig. 5C). PCA and multidimensional scaling (MDS) of gene expression profiles showed obvious differences between the two groups (Fig. 5D). In the gene set enrichment analysis (GSEA), hallmark OXPHOS, hallmark fatty acid metabolism, and KEGG one-carbon pool by folate were significantly upregulated in patients with high *ALDH1L1* expression compared with those with low expression (Fig. 5E). These data are consistent with our findings in HuH-7 cells and imply that investigation of *ALDH1L1* expression could be a useful therapeutic strategy for HCC.

## Discussion

In this study, we demonstrated that genetic modulation of the folate cycle affects not only one-carbon metabolism but also other related metabolism systems, and influences mitochondrial activity and ZMP sensitivity. *ALDH1L1* expression is usually reduced or diminished in HCC, and previous studies suggested that *ALDH1L1* is a tumor suppressor gene^13–17^. Thus, our *ALDH1L1*-expressing HuH-7 cell line model was suitable for analyzing the relationship between one-carbon metabolism and tumor progression. Namely, H7-emp cells reflect a more malignant state of tumor cells than H7-1L1 cells.

Under normal cell growth conditions, there were no differences in proliferation rates and cellular morphology between H7-emp and H7-1L1 cells. However, metabolome analysis clearly revealed that some metabolites that associate with one-carbon metabolism, choline metabolism, and *dn*PS significantly varied with or without ALDH1L1 expression. Because decreased Ser and increased Gly were observed in H7-1L1 cells, ALDH1L1 might positively drive one-carbon metabolism. Related to this, it is speculated that 5-CH_3_-THF is utilized for Met synthesis in H7-1L1 cells, although homocysteine receives a methyl group from 5-CH_3_-THF or betaine. Supporting this idea, the increase in phosphocholine and glycerophosphocholine in H7-1L1 cells might be due to suppression of the choline–betaine–dimethylglycine synthesis pathway. This is consistent with a previous report showing that dimethylglycine is accumulated in *Aldh1l1*-deficient mice^18,20^. It is known that a Met- and choline-deficient diet is a conventional and useful rodent model to induce NASH, which is a risk factor of HCC^24^. Met and choline are essential for hepatic ß-oxidation and the production of very low-density lipoprotein (VLDL). Therefore, a Met- and choline-deficient diet impairs ß-oxidation, resulting in ROS production to induce steatohepatitis. Our findings and the results from the *Aldh1l1*-deficient mice study support that ALDH1L1 promotes choline metabolism and suppresses progression to NASH. Importantly, ZMP, a *dn*PS intermediate, was accumulated in H7-1L1 cells. Cytosolic 10-fTHF is a co-substrate for ALDH1L1, MTHFD1, GART, and ATIC. Because 10-fTHF is consumed by highly expressed ALDH1L1, *dn*PS might be suspended at the point of ATIC’s formyl group transfer reaction. As a result, ZMP accumulation as well as *dn*PS arrest is reasonable for tumor suppression because tumor cells require numerous nucleotides that are supplied from *dn*PS and/or the salvage pathway for replication of DNA.

**Figure 6:**
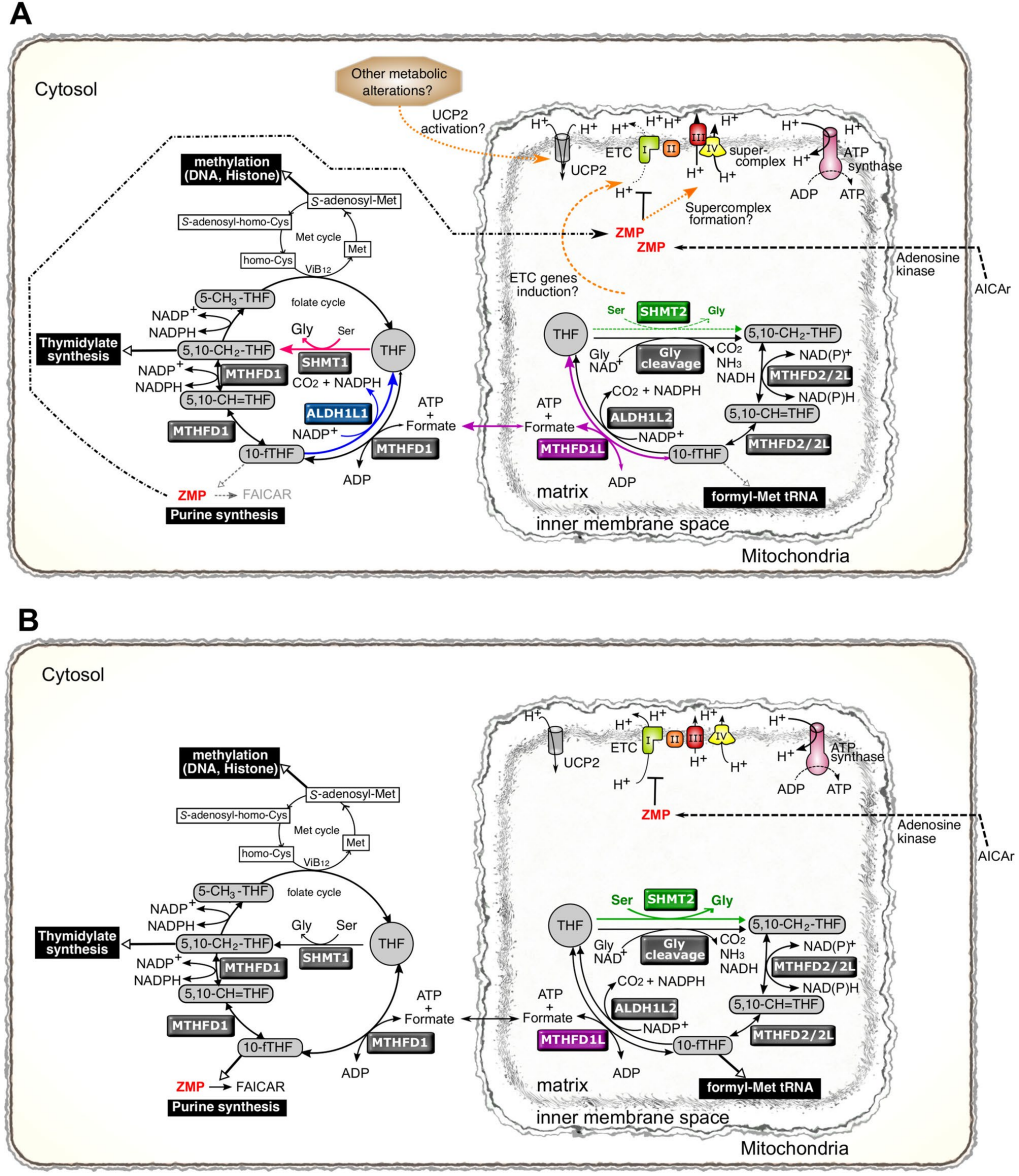
Schematic diagram of the effects of ALDH1L1 and ZMP on the folate cycle and mitochondria. Model postulating how ALDH1L1 could suppress cancer cell promotion. (**A**) In an *ALDH1L1*-expressing cell, ALDH1L1 predominantly consumes 10-fTHF. The transfer reaction of a formyl group from 10-fTHF required for the synthesis of FAICAR is impaired by the consumption of 10-fTHF, resulting in the accumulation of ZMP. ZMP inhibits ETC complex I independent of AMPK, resulting in decreased mitochondrial spare capacity. Whereas ETC supercomplex composed of complex III and IV might be assembled by ZMP, leading to higher mitochondrial membrane potential. At the same time, THF generated in the cytoplasm is catalyzed by SHMT1 to activate folate cycle, resulting in a decrease in intracellular Ser levels. Mitochondria folate cycle is expected to be activated to supply formate by MTHFD1L to the cytoplasm and to accelerate the reaction of 10-fTHF synthesis by MTHFD1 in the cytoplasm. Synthesis of 5,10-CH_2_-THF from THF in mitochondria is thought to proceed mainly by Gly cleavage, and not SHMT2, because of the decreased intracellular Ser levels. ETC genes are probably induced by functional suppression of SHMT2. Mitochondrial proton gradient is counteracted by ATP synthase, UCP2, and others. Metabolism alterations induced by ALDH1L1 may activate UCP2 or other proton leakage machineries. (**B**) In a malignant tumor cell without *ALDH1L1*, 10-fTHF is utilized by ZMP in an FAICAR-generating reaction, resulting in enhancement of *dn*PS, which is suitable for cancer cell proliferation. Exogenous AICAr is converted to ZMP and inhibits complex I. The insufficient amount of ZMP compared with that in *ALDH1L1*-expressing cells may result in insufficient supercomplex formation and reduced mitochondrial membrane potential.

Mitochondrial Ser catabolism may contribute to ZMP accumulation. Similar to cytosolic THF, SHMT2 catalyzes mitochondrial THF to 5,10-CH_2_-THF accompanied by Ser to Gly conversion. In addition to this reaction, it is known that the Gly cleavage system can catalyze mitochondrial THF to 5,10-CH_2_-THF. Mitochondrial THF could be converted by both reactions in H7-emp cells (Fig. 6B). However, in H7-1L1 cells, this conversion may have to rely on the Gly cleavage system because intracellular Ser levels are reduced (Fig. 1, 6A). Furthermore, cytosolic 10-fTHF may have to be supplied from mitochondria by mediating formate transfer because cytosolic 10-fTHF is consumed by ALDH1L1. Although MTHFD1 synthesizes 10-fTHF from THF and formate in the cytosol, the mitochondrial folate cycle must be activated to supply formate to cytosol. Interestingly, Tong et al.^25^ reported that knockdown of *SHMT2* caused ZMP accumulation. Similar to our observations, arrest of the mitochondrial folate cycle may cause impairment of formate transfer from mitochondria to the cytosol, resulting in cytosolic 10-fTHF reduction and ZMP accumulation. In other words, ALDH1L1 also disrupts Ser catabolism in mitochondria, which may contribute to an additive increase in ZMP.

Mitochondrial membrane potential is usually composed by the balance of proton gradients. JC-1 analysis showed that the proportion of the mitochondrial membrane potential was higher in H7-1L1 cells than in H7-emp cells even in the absence of AICAr, whereas the proportion of the spare capacity was lower in H7-1L1 cells. These observations indicate that ALDH1L1 indirectly regulates mitochondrial OXPHOS because ALDH1L1 localizes in the cytosol. As mentioned above, ZMP was accumulated in H7-1L1 cells. Therefore, there is a possibility that OXPHOS is regulated by intracellular ZMP levels. It is well known that AMPK is a regulator of energy balance that promotes catabolic processes and inhibits anabolic processes. Although ZMP is an activator of AMPK, it assumes that AMPK contribution is comparable between H7-emp and H7-1L1 cells because there was no significant difference in AMPK phosphorylation levels. Meanwhile, Guigas et al.^26^ reported that AICAr inhibits hepatocyte OXPHOS, which is AMPK-independent. Importantly, they suggested that ZMP inhibits the ETC complex I. Therefore, *ALDH1L1* expression-induced ZMP accumulation might suppress ETC complex I. It is reported that the spare capacity is dependent on the ETC complex I in certain cell types^27,28^. This seems consistent with the fact that accumulated ZMP inhibits ETC complex I, resulting in reduced spare capacity in H7-1L1 cells. The fact that mitochondrial membrane potential remains high in H7-1L1 cells despite the reduced spare capacity is presumably due to alternative expression and/or activation of other ETC complexes. In recent years, the existence of assemblies of the ETC complexes, called supercomplexes, which facilitate effective electron transfer, has been recognized in ETC. Hofer et al. reported that AICAr accelerates the formation of ETC supercomplexes, especially the assembly of complex III and IV (SC-III + IV) was obviously increased in HeLa cells^29^. Although it is unknown whether AICAr inhibits ETC complex I in this situation, if the SC-III + IV is active, higher mitochondrial membrane potential in H7-1L1 cells might be caused by the formation of the SC-III + IV. (Fig. 6A).

In our observations, AICAr sensitivity differed between H7-emp and H7-1L1 cells. In both cell lines, the cell cycle was arrested at S phase and suppressed cell proliferation (Fig. 2A–C). Nevertheless, mitochondrial activity in H7-1L1 cells was higher than in H7-emp cells, resulting in more resistance to AICAr (Figs. 3, 4). In particular, ALDH1L1 expression seemed to sustain generation of the proton gradient and mitochondrial membrane potential. This is also supported by the observation that mitochondrial ROS was higher in H7-1L1 cells than in H7-emp cells because mitochondria with high ECT activity produce ROS^30^ (Fig. 4G). According to previous studies, *SHMT2* knockdown HeLa cells and *SHMT2* knockout 293A cells had upregulated ETC complex gene expression^25,31^. Although SHMT2 was expressed to the same extent in both H7-emp and H7-1L1 cells (Supplemental Fig. 2), it might be functionally suppressed due to decreased Ser in H7-1L1 cells. Thus, ALDH1L1 may induce expression of ETC complex genes indirectly and generate the proton gradient. Whereas *SHMT2* knockout Jurkat and HCT116 cells and *Shmt2*-deficient murine embryonic fibroblast cells showed reduced expression of ETC complex genes^32–34^. The regulatory mechanisms are still controversial and remain unresolved.

It is known that the proton gradient is offset by the action of ATP synthase and proton leak, which is partly mediated by UCPs. The ATP-linked respiration was suppressed by AICAr in both H7-emp and H7-1L1 cells (especially when treated for 24 h), whereas the proton leak was reduced in H7-emp cells and maintained in H7-1L1 cells (Fig. 3). These results indicate that ALDH1L1 indirectly induces mitochondrial membrane potential as well as proton leak to counteract the proton gradient. There are five UCP genes in mammals. Apart from *UCP2*, the expression of other UCPs is comparatively restricted: *UCP1* is normally expressed in brown adipose tissue, *UCP3* in muscle, and *UCP4* and *UCP5* in neurons, while *UCP2* is expressed ubiquitously^30^. Thus, *UCP2* is likely involved in proton leak in HuH-7 cells. It is known that *UCP2* is regulated by gene expression and activation mechanisms. ROS are well known UCP2 inducers, and upregulated *UCP2* acts as an indirect eliminator of ROS^30,35^. Despite higher ROS levels in H7-1L1 cells, there was no difference in UCP2 expression between H7-emp and H7-1L1 cells, indicating that the resolution of mitochondrial membrane potential was independent of UCP2 expression (Fig. 4G, Supplemental Fig. 2). UCP2 is activated by polyunsaturated fatty acid and 4-hydroxynonenal and inhibited by purine nucleotides such as ATP^36^. UCP2 activation may be caused by certain metabolites as a result of metabolism changes by ALDH1L1. Alternatively, other factors associated with proton leak, such as adenine nucleotide translocase expression, may be involved in this phenotype^37^.

In line with our observations in HuH-7 cells, liver cancer cell lines with lower expression of *ALDH1L1* were ZMP-sensitive (Fig. 5A). It is worth mentioning that cordycepin, which is an AMP analog, was the most sensitive compound within the PRISM repurposing primary screen data set in the lower *ALDH1L1*-expressing liver cancer cell lines (Fig. 5B). Furthermore, consistent with our findings, GSEA analysis revealed that OXPHOS-, fatty acid metabolism-, and folate metabolism-related genes are preferentially enriched in HCC with higher expression of *ALDH1L1*. In the clinic, AICAr is utilized for tumors such as B cell chronic lymphocytic leukemia, mantle cell lymphoma, hematological cancers, and cervical cancer^38^. In addition, cordycepin is undergoing clinical trials^38^. Our findings suggest that AICAr, cordycepin, and possibly adenosine analogs are candidate drugs for anti-HCC with lower or no *ALDH1L1* expression. Recent clinical studies indicated that *ALDH1L1* expression is a candidate prognostic biomarker of hepatitis B virus-related HCC patients (i.e., patients with lower expression of *ALDH1L1* show shorter survival)^16^. Therefore, we believe that investigation of *ALDH1L1* expression levels will allow us to predict prognosis, and the use of these drugs in patients with low or no *ALDH1L1* expression is effective in improving prognosis.

## Methods

### Reagents

5-Aminoimidazole-4-carboxamide riboside (AICAr) was purchased from Fujifilm Wako Pure Chemical (Osaka, Japan). A-769662 and carbonyl cyanide 3-chlorophenylhydrazone (CCCP) were purchased from Tokyo Chemical Industry (Tokyo, Japan).

### Cell culture

HuH-7 cells were obtained from the Japanese Collection of Research Bioresources (JCRB) cell bank. HuH-7 cells were cultured in Dulbecco’s modified Eagle medium (DMEM) (Nacalai Tesque Inc., Kyoto, Japan) supplemented with 5% fetal bovine serum (FBS; Thermo Fisher Scientific, Waltham, MA, USA), and 1 × antibiotic–antimycotic mixed solution (Nacalai Tesque) at 37 °C in a 5% CO_2_ incubator.

### DNA oligonucleotides

DNA oligonucleotides used in this study were synthesized by and purchased from Fasmac (Kanagawa, Japan) or Integrated DNA Technologies (Coralville, IA, USA). The sequences of synthesized oligonucleotides are shown in Supplementary Table 1.

### Virus production

Lentivirus and packaging plasmids CSII-CMV-MCS-IRES2-Bsd and pCAG-HIVgp and pCMV-VSV-G-RSV-Rev were provided by the RIKEN BRC through the National BioResource Project of the MEXT, Japan. Human *ALDH1L1* cDNA was amplified by PCR (corresponding primers are shown in Supplementary Table 1) from Human BioBank cDNA (Primer Design, UK), cloned into pBluescriptII SK(+) plasmid, and then sub-cloned into CSII-CMV-MCS-IRES2-Bsd lentiviral vector. Lentivirus particles were produced by transfection of CSII-CMV-MCS-IRES2-Bsd or CSII-CMV-ALDH1L1-IRES2-Bsd, pCAG-HIVgp and pCMV-VSV-G-RSV-Rev into HEK293 cells, which were subsequently used to infect to HuH-7 cells. Transduced HuH-7 cells were selected with 5 mg/mL blasticidin S hydrochloride (Fujifilm Wako Pure Chemical).

### Real-time RT-PCR quantification of mRNA

Total RNA was extracted and purified using Sepasol-RNA I Super G (Nacalai Tesque) according to the manufacturer’s protocol. Purified RNA was reverse-transcribed using ReverTra Ace (Toyobo, Osaka, Japan). cDNAs were subjected to quantitative real-time PCR using THUNDERBIRD SYBR qPCR Mix (Toyobo). Primers used for real-time qPCR analysis are listed in Supplemental Table 1. Real-time PCR was performed in triplicate using a StepOnePlus Real-Time PCR System (Thermo Fisher Scientific). Relative expression ratios were calculated using the ΔΔCt method. TATA box binding protein (*TBP*) was used as a normalization reference for determining target gene expression levels, and Human BioBank cDNA was used as calibrators in each experiment.

### Measurement of mitochondrial respiration capacity

HuH-7 cells and their derivatives were plated in a Seahorse XF96 Cell Culture Microplate (Agilent Technologies, Santa Clara, CA, USA) at a density of 12,000 cells in 80 μL DMEM supplemented with 5% FBS and antibiotics per well and were incubated overnight at 37 °C in 5% CO_2_. The culture medium was replaced with XF DMEM (Agilent) supplemented with glucose (10 mM), L-glutamine (2 mM), and sodium pyruvate (1 mM) at pH 7.4, and cells were further incubated at 37 °C in a non-CO_2_ incubator for 1 h. The OCR and the extracellular acidification rate (ECAR) were measured using the Agilent Seahorse XFe96 Analyzer and mitochondrial reparation was assessed by sequential addition of 1.5 μM oligomycin (Agilent), 0.5 μM fluoro-carbonyl cyanide phenylhydrazone (Agilent), and 1.0 μM rotenone and antimycin A (Agilent), according to the manufacturer’s method. After analysis, cells were fixed in 70% ethanol and stained with Hoechst 33,258 to count the remaining cells. Cell numbers were calculated from fluorescent images processed with Image-J software and used to normalize OCR and ECAR data.

### Metabolome analysis

Metabolome analysis was performed at Human Metabolome Technologies (HMT, Yamagata, Japan). Intracellular metabolites were extracted according to the manufacturer’s protocol. Metabolome analysis was performed by capillary electrophoresis time-of-flight mass spectrometry (CE-TOFMS). Metabolite peaks were quantified and normalized according to cell number.

### Immunoblotting

Cell lysates prepared in lysis buffer [50 mM Tris–HCl (pH 7.6), 150 mM NaCl, 1%(v/v) Triton X-100, 0.1% (w/v) sodium dodecyl sulfate (SDS), 1 × Protease Inhibitor Cocktail (Nacalai Tesque)] were electrophoretically separated on denaturing polyacrylamide gels containing 0.1% (w/v) SDS and transferred to PVDF membrane membranes (Pall, Port Washington, NY, USA) in transfer buffer containing 10% methanol. Membranes were probed with primary antibodies. A detailed description of the primary antibodies and blocking conditions is provided in Supplementary Table 2. Sites of antibody binding were detected using HRP-conjugated antibodies against rabbit IgG (1:10,000; Promega, Madison, WI, USA), mouse IgG (1:10,000; Jackson ImmunoResearch, West Grove PA, USA), or mouse IgM (1:2000; Tokyo Chemical Industry). Immunocomplexes were visualized by Immobilon Western Chemiluminescent HRP substrate (Merck Millipore, Burlington, MA, USA) or Pierce ECL Western Blotting Substrate (Thermo Fisher Scientific).

### Cell cycle analysis

Cell cycle was determined by flow cytometry of cells labelled with anti-BrdU-FITC and DAPI. Asynchronously growing cells were pulse-labelled with 10 µM 5- bromo-2′-deoxyuridine (BrdU) (Nacalai Tesque) for 3 h prior to harvesting. Cells were trypsinized and fixed in ice-cold 70% ethanol for 30 min on ice. Cells were suspended in 2 M HCl containing 0.5% (w/v) Triton X-100 for 30 min at room temperature. After supernatant was discarded, cells were resuspended in 0.1 M Na_2_B_4_H_7_ (pH 8.5) for 2 min at room temperature. Cells were then washed in buffer (1% bovine serum albumin (BSA) and 0.05% Tween 20 containing PBS) and treated with FITC-conjugated anti-BrdU antibody (BioLegend, San Diego, CA, USA) overnight at 4 °C. Cells were washed with 1% BSA containing PBS, centrifuged, and resuspended in 10 µg/mL 4′,6-diamidino-2-phenylindole (DAPI) (Nacalai Tesque), and allowed to incubate for 30 min on ice. Cell cycle analysis was performed on the Attune NxT flow cytometer (Thermo Fisher Scientific).

### Cell viability assay

MTS [3-(4,5-dimethylthiazol-2-yl)-5-(3carboxymethoxyphenyl)-2-(4-sulfophenyl)-2H-tetrazolium, inner salt] assay was performed with a CellTiter 96 AQ_ueous_ Non-Radioactive Cell Proliferation Assay (Promega). To prepare MTS/PMS solution, one volume of 3.15 mM phenazine methosulfate (PMS) (Nacalai Tesque) solution was mixed with 20,000 volume of 2 mg/L MTS solution. MTS/PMS solution was diluted into cell culture medium at a concentration of 15%, and then 100 µL 15% MTS/PMS medium was added to the wells. Cell plates were incubated at 37** °C** for 1 to 3 h until the absorbance at 490 nm was approximately 1. Absorbance was measured using an SH-1300Lab microplate reader (Hitachi High-Tech, Tokyo, Japan). Absorbance was normalized to and expressed as a relative percentage of the plate-averaged solvent control.

### High-content analysis

All cells were seeded on a 96-well imaging plate (PerkinElmer, Waltham, MA, USA) and analyzed by the Operetta CLS high-content analysis system (PerkinElmer). For mitochondrial ROS measurement, cells were incubated with mtSOX deep red (Dojindo Laboratories, Kumamoto, Japan) according to the manufacturer’s protocol. For mitochondrial membrane potential (MMP) measurement, cells were incubated with 2 µM JC-1 probe (Dojindo Laboratories) for 30 min at 37 °C and then washed with JC-1 imaging buffer. For both ROS and MMP measurements, cells were stained with 1 drop/mL Hoechst 33,342 (Thermo Fisher Scientific), and each fluorescent intensity was normalized by the number of Hoechst 33,342-positive nuclei. mtSOX deep red was excited at 530–560 nm, monomeric JC-1 at 460–490 nm, aggregated JC-1 (J-aggregates) at 530–660 nm, and Hoechst 33,342 at 355–385 nm.

### Live cell imaging

HuH-7 cells were seeded on 35 mm glass bottomed dishes (Matsunami glass Industry, Osaka, Japan) for 24 h. Cells were incubated with 2 µM JC-1 probe (Dojindo Laboratories) for 30 min at 37 °C and then washed with JC-1 imaging buffer. Images were acquired with the Zeiss LSM 900 Airyscan 2 super-resolution system (Carl Zeiss, Oberkochen, Germany). Microscopes were equipped with an environmental chamber that maintained 37 °C with humidified 5% CO_2_ gas during imaging.

### Drug sensitivity data analysis

Correlations between *ALDH1L1* gene expression and drug sensitivity in 18 liver cancer cell lines were retrieved from DepMap using the 22Q4 public data set for gene expression, Sanger GDSC1 drug sensitivity replicate-level dose data set, and PRISM repurposing primary screen drug sensitivity dataset for drug response data (https://depmap.org/portal/. Accessed on 23 Feb 2023).

### Gene expression analysis

Normalized gene expression data were obtained from Pan-Cancer Atlas (TCGA)^39^ for liver HCC patients. GSEA was performed to compare the following groups: patients with high *ALDH1L1* expression (n = 20, Z-score > 1.2) versus those with low *ALDH1L1* (n = 12, Z-score <  − 1.2)^40^. The analyses were performed using the HALLMARK and KEGG gene sets. The normalized enrichment score NES was calculated by GSEA software. A false discovery rate q-value of < 0.25 was considered significant. PCA and MDS were conducted using iDEP^41^.

### Supplementary Information

Supplementary Information 1.

Supplementary Information 2.

## Data Availability

The data that support the findings of this study are presented in this published article, its supplementary data. Metabolomics data were deposited in the Metabolomics Workbench^42^. Accession code: ST002584.
