# Supplementary material for: One-carbon metabolizing enzyme ALDH1L1 influences mitochondrial metabolism through 5-aminoimidazole-4-carboxamide ribonucleotide accumulation and serine depletion, contributing to tumor suppression
Source: Sci Rep. 2023 Aug 18;13:13486. doi: 10.1038/s41598-023-38142-5 (PMC10439146; doi:10.1038/s41598-023-38142-5)
Supplement: Supplementary file 1 — Supplementary Information 1. [file 41598_2023_38142_MOESM1_ESM.pdf]

One-carbon metabolizing enzyme ALDH1L1 influences mitochondrial metabolism through 5-aminoimidazole-4-carboxamide ribonucleotide accumulation and serine depletion, contributing to tumor suppression

Masato Sasaki[1]*, Kazuo Yamamoto[2], Takeshi Ueda[3,4], Hayato Irokawa[5], Kouki Takeda[5], Ryoya Sekine[5], Fumie Ito[1], Yutaka Tanaka[1], Shusuke Kuge[5], Nobuyuki Shibata[1]

[1] Division of Infection and Host Defense, Faculty of Pharmaceutical Sciences, Tohoku Medical and Pharmaceutical University, 4-4-1, Komatsusima, Aoba-ku, Sendai, Miyagi 981-8558, Japan.

[2] Biomedical Research Support Center, Nagasaki University School of Medicine, 1-12-4 Sakamoto, Nagasaki, 852-8523, Japan.

[3] Department of Biochemistry, Kindai University Faculty of Medicine, Osakasayama, Osaka 589-8511, Japan.

[4] Graduate School of Medical Sciences, Kindai University Faculty of Medicine, Osakasayama, Osaka 589-8511, Japan.

[5] Division of Microbiology, Faculty of Pharmaceutical Sciences, Tohoku Medical and Pharmaceutical University, 4-4-1, Komatsusima, Aoba-ku, Sendai, Miyagi 981-8558, Japan.

*Correspondence and requests for materials should be addressed to M.S.
Phone: +81-22-727-0132
email: msasaki@tohoku-mpu.ac.jp

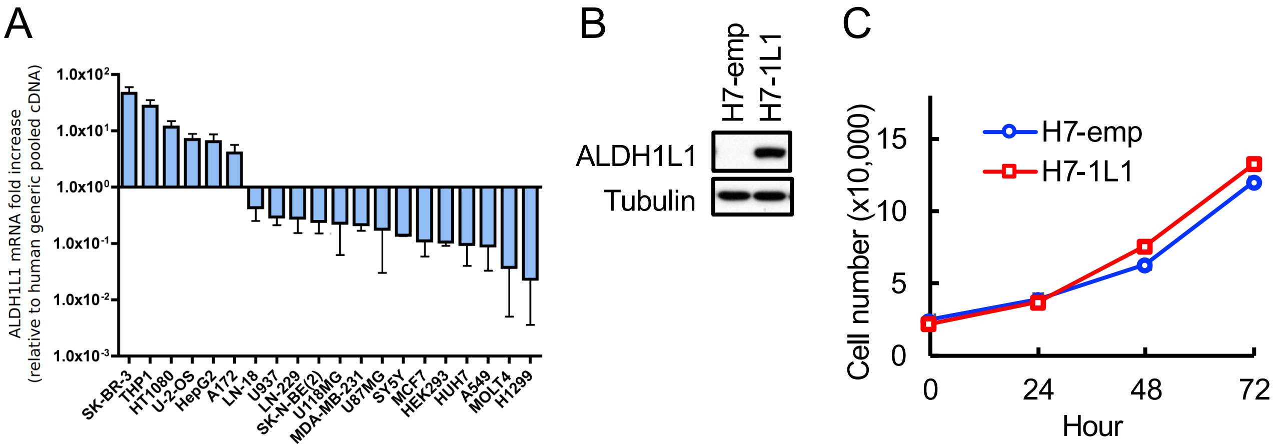

Supplemental Figure 1. ***ALDH1L1*** **expression in human cancer cell lines and proliferation rate of** ***ALDH1L1*****-expressing HuH-7 cells.** (**A**) Comparison of *ALDH1L1* mRNA expression in human cancer cell lines. Data are expressed as the mean fold difference between pooled human cDNA (set to 1) and cDNA of each cell line after normalization to *TBP* mRNA expression. Results are the mean ± SEM of triplicates. (**B**) ALDH1L1 expression in control (H7-emp) or *ALDH1L1*-expressing lentivirus-infected HuH-7 (H7-1L1) cells. (**C**) Proliferation curves of H7-emp and H7-1L1 cells cultured in complete medium. Results are the mean ± SEM of triplicates.

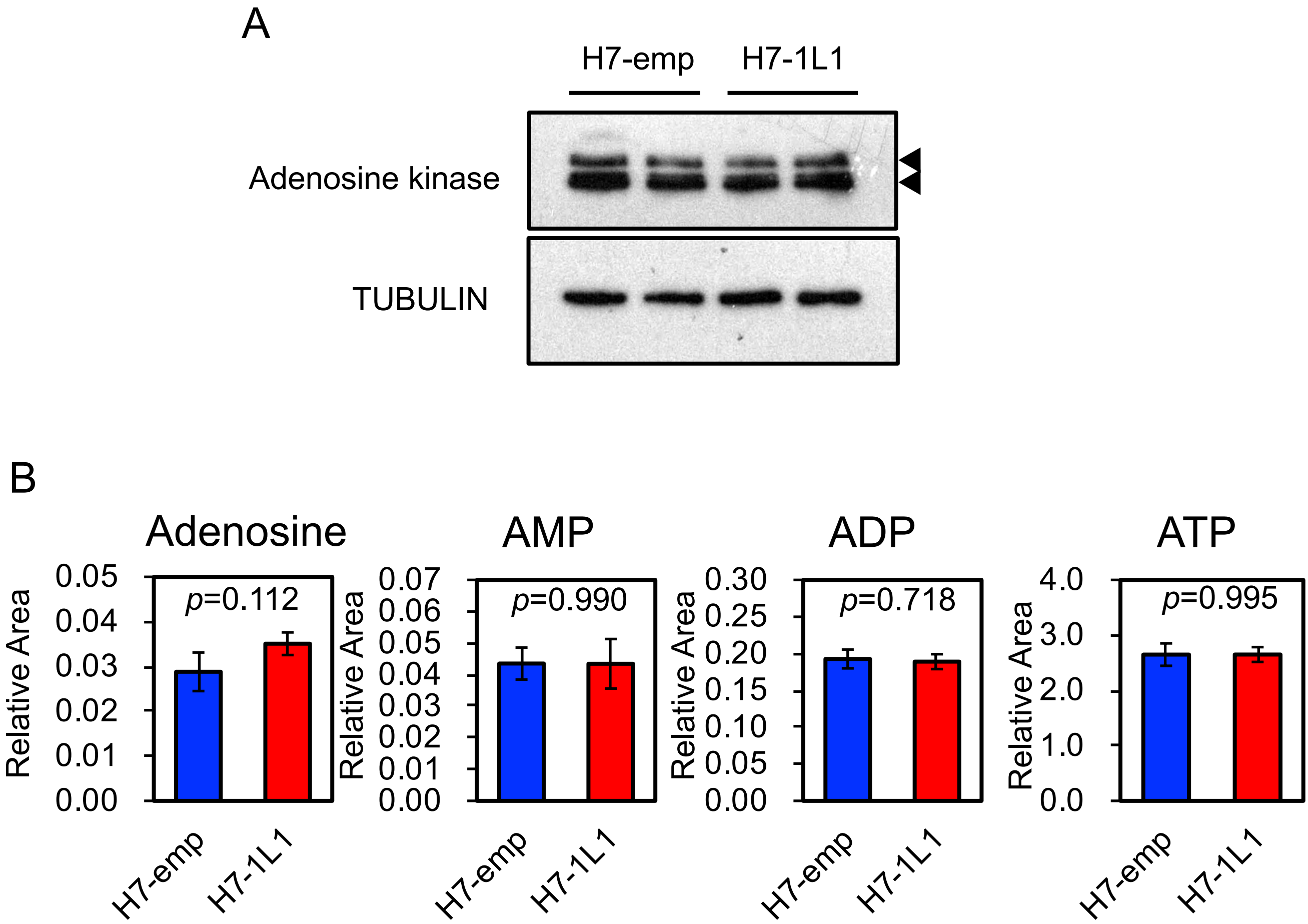

Supplemental Figure 2. Adenosine and its nucleotides levels affecting adenosine kinase activity.

(A) Protein levels of adenosine kinase and tubulin were measured in H7-emp and H7-1L1 cells by western blotting.

(B) Levels of adenosine, and its nucleotides, are not significantly different in both H7-emp and H7-1L1 cells. Results are the mean ± SEM of triplicates; *p*-values, unpaired Student's *t*-test.

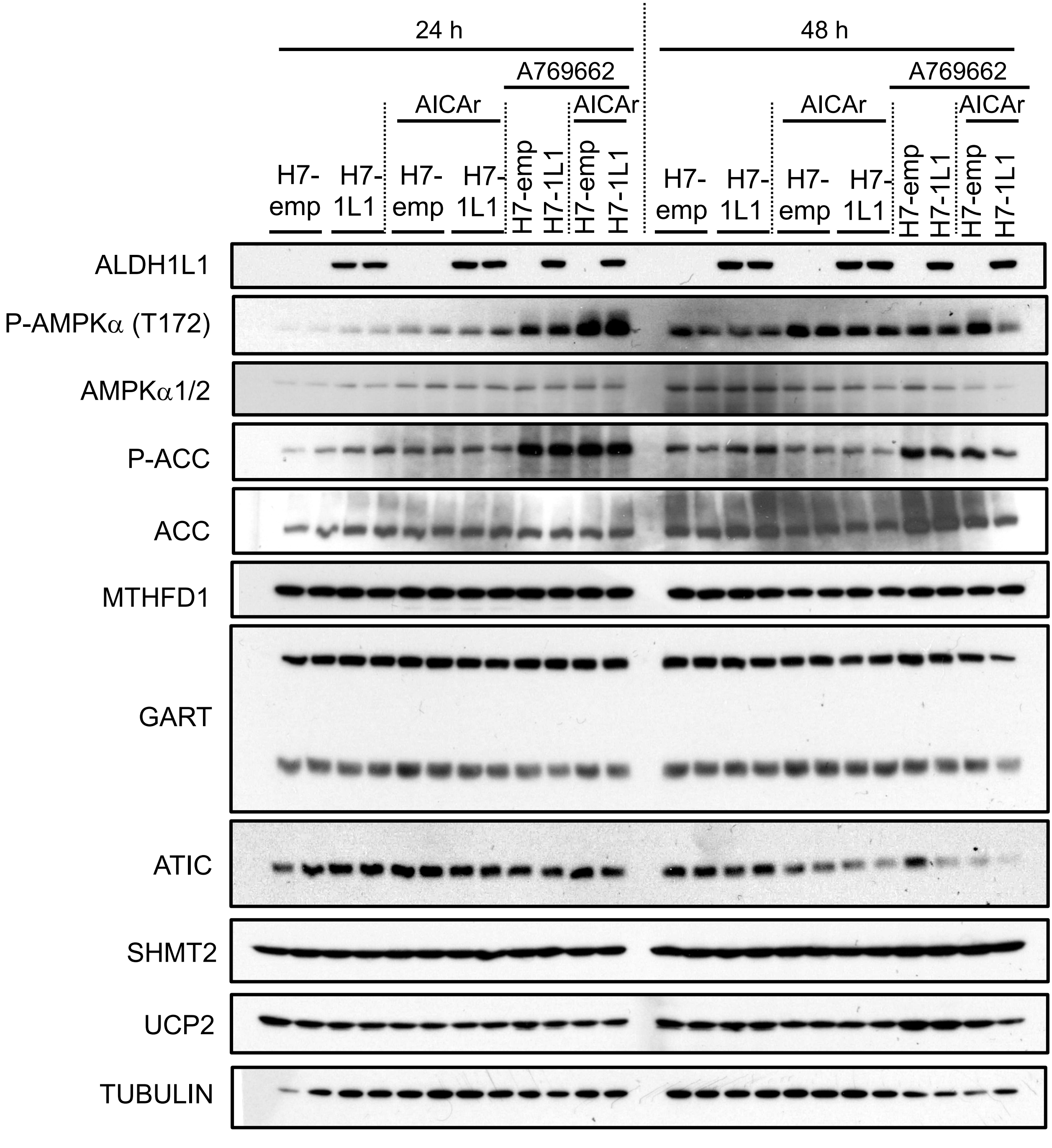

Supplemental Figure 3. AMPK activation and expression of 10-fTHF-metabolizing enzymes. Protein levels of ALDH1L1, phosphorylated AMPKα, AMPKα1/2, phosphorylated ACC, ACC, MTHFD1, GART, ATIC, SHMT2, UCP2, and tubulin were measured in H7-emp and H7-1L1 cells by western blotting. The cells were treated with 0.5 mM AICAr and 0.1 mM A-769662 for 24 h (left) or 48 h (right).

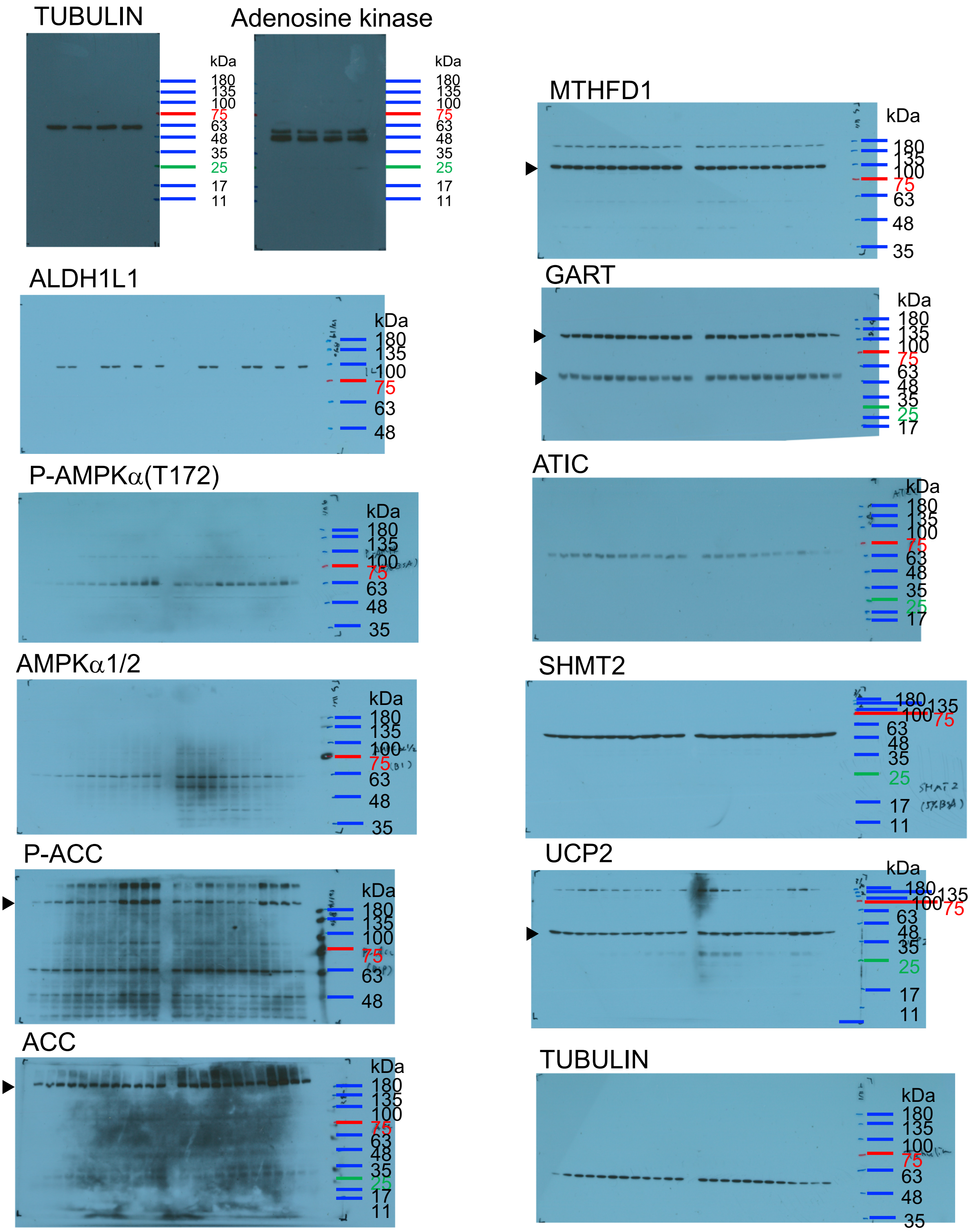

Supplemental Figure 4. Original full-size blot images used to generate panels in Supplemental Figure 2A and 3. Molecular mass standards were from GeneDireX BlueRAY prestained protein ladder.

Supplemental table 1 Primers used for cloning and quantitative PCR

Primer	Sequence
EcoRI-Kozac-1L1_Fw	5'-GAATTCCCGCCGCCACCATGAAGATTGCAGTGATTGGACAGAG-3'
NotI-1L1-Cter_Rv	5'-GCGGCCGCTCAGTATTCGAAGGTCACTG-3'
1L1 qPCR(1580-1600)_Fw	5'-TCCAGACCTTCCGCTACTTTG-3'
1L1 qPCR(1731-1711)_Rv	5'-CAGGGGATAGTTCCAGGGGAT-3'
TBP qPCR_Fw	5'-CCCGAAACGCCGAATATAATCC-3'
TBP qPCR_Rv	5'-CACGAACCACGGCACTGATT-3'

Supplemental table 2 List of primary antibodies used for immunoblotting assays

Target protein	Source	Cat#	Dilution	Blocking
ALDH1L1	Proteintech (Rosemont, IL, USA)	17390-1-AP	1 : 1,000	2% nonfat milk
Adenosine kinase (ADK)	Proteintech	66929-1-Ig	1 : 5,000	Blocking One (Nacalai Tesque)
phospho-AMPKα (Thr172) (40H9)	Cell Signaling Technology (Danvers, MA, USA)	#2535	1 : 1,000	5% BSA
AMPKα/β (D-6)	Santa Cruz Biotechnology (Dallas, TX, USA)	sc-74461	1 : 500	Blocking One (Nacalai Tesque)
phospho-acetyl-CoA carboxylase (Ser79)	Cell Signaling Technology	#3661	1 : 1,000	Blocking One-P (Nacalai Tesque)
acetyl-CoA carboxylase	Cell Signaling Technology	#3662	1 : 1,000	2% nonfat milk
AICAR transformylase (F38 P7 H9) <ATIC>	Santa Cruz Biotechnology	sc-53612	1 : 500	2% nonfat milk
GART (D-4)	Santa Cruz Biotechnology	sc-166379	1 : 500	2% nonfat milk
MTHFD1 (A-8)	Santa Cruz Biotechnology	sc-271412	1 : 500	2% nonfat milk
mSHMT (F-11) <SHMT2>	Santa Cruz Biotechnology	c-390641	1 : 500	5% BSA
UCP2 (G-6)	Santa Cruz Biotechnology	sc-390189	1 : 500	2% nonfat milk
α-tubulin	Fujifilm Wako Pure Chemical (Osaka, Japan)	10G10	1 : 5,000	2% nonfat milk
